# *Raphia vinifera* (Arecaceae; Calamoideae): Misidentified for far too long

**DOI:** 10.3897/BDJ.7.e37757

**Published:** 2019-08-27

**Authors:** Suzanne Mogue Kamga, Bonaventure Sonké, Thomas L.P. Couvreur

**Affiliations:** 1 Université de Yaoundé I, Ecole Normale Supérieure, Yaoundé, Cameroon Université de Yaoundé I, Ecole Normale Supérieure Yaoundé Cameroon; 2 Institut de Recherche pour le Développement (IRD), Montpellier, France Institut de Recherche pour le Développement (IRD) Montpellier France

**Keywords:** *Raphia mambillensis*, synonymy, Palisot de Beauvois, Africa, taxonomy, *R. mannii*

## Abstract

**Background:**

The genus *Raphia* (Arecaceae/Palmae) is the most economically and culturally important genus of African palms. With over 20 recognized species, it is also the most diversified among tropical African palms. However, significant taxonomic confusion still persists in the genus. *Raphia vinifera* P.Beauv. is one of the first two names described in the genus, but the species has been misidentified and confused for several decades.

**New information:**

We clarify the taxonomic identity of *R. vinifera.* We retrace the taxonomic history of the name and clarify its morphological identity using the literature and type material. We synonymize the name *R. mambillensis* with *R. vinifera.* We provide a new detailed morphological description of *R. vinifera* based on the study of herbarium material and field data. A distribution map is also provided. *Raphia vinifera* is still incompletely known, and more research should be undertaken on this species' presence and ecology, especially in West Africa.

## Introduction

### Taxonomic history of the name *Raphia vinifera*

The palm genus *Raphia* P.Beauv. contains 22 species (Mogue Kamga et al. 2018) mainly distributed in continental Africa, with one species reaching Madagascar and one endemic species in Central and South America (Dransfield et al. 2008, Stauffer et al. 2014). This genus is also one of the most widely used and socio-economically important across Africa (Obahiagbon 2009, Tuley 1995, Burkill 1985).

**Figure 1.**
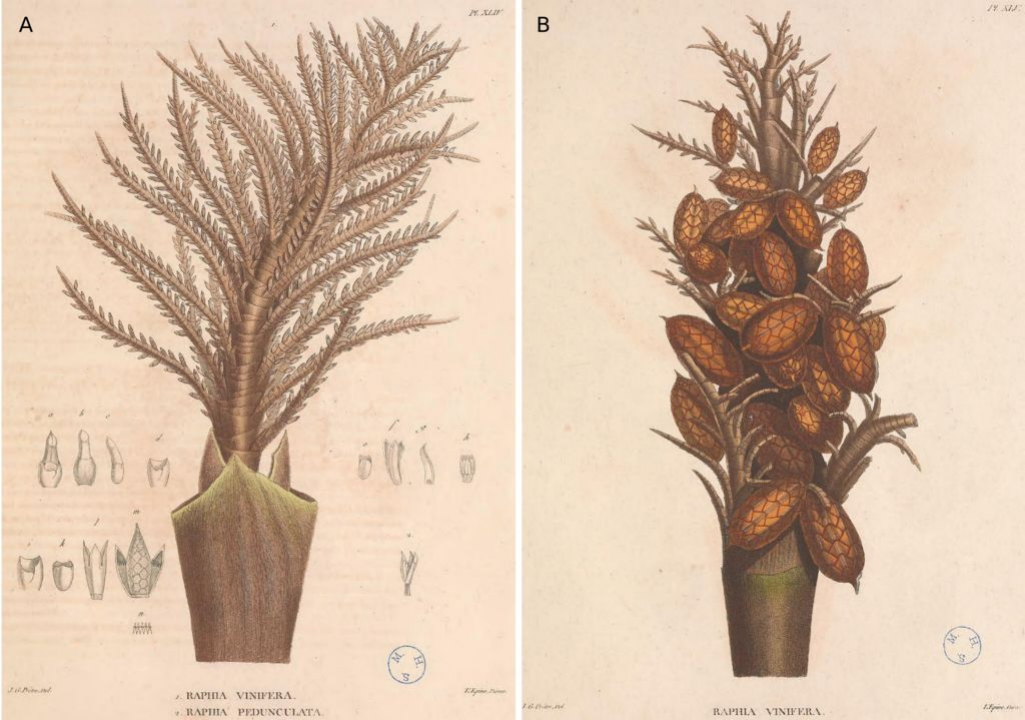
Illustrations of *Raphia vinifera* from Palisot de Beauvois (1804), taken from “Flore d’Oware et de Benin”. **A** Inflorescence with young flowers (https://www.biodiversitylibrary.org/item/181617#page/183/mode/1up); **B** Infructescence (https://www.biodiversitylibrary.org/item/181617#page/185/mode/1up). Note the difference in inflorescence structure.

*Raphia* was erected by Palisot de Beauvois (1804), who recognized the monoecious nature of the *Raphia* inflorescences, thus separating it from the lianescent and mainly Asian genus *Calamus* L. (rattans, dioecious) to which it was tentatively associated by Jussieu in Jussieu and Usteri (1791). Palisot de Beauvois (1804) described two new species: *Raphia pedunculata* P.Beauv. from Madagascar (now *R. farinifera* (Gaertn.) Hylander) and *Raphia vinifera* P.Beauv. from West Africa. The latter was suggested to be common along the rivers of the Oware (now in Delta State) and Benin (now in Edo State) provinces in nowadays south-eastern Nigeria. No collection number was mentioned in the protologue. However, the new name was accompagnied by a short description and two illustrations with analysis (code Art. 38.7, Turland et al. 2018) and is thus validely published (Art. 38.8): one of a flowering partial inflorescence and one of a fruiting partial inflorescence (Fig. 1). These drawings, however, appear to belong to two different species, being quite different in their morphology. Indeed, following the terminology of Otedoh (1982), the drawing of the partial fruiting inflorescence (Fig. 1B) is “raphiate”, being non-planar with widely interspaced rachillae (lax) and inconspicuous bracts, while the drawing of the flowering inflorescence (Fig. 1A) is distinctly “flabellate”, being planar with tightly compressed rachillae and large conspicuous bracts. In addition, *R. vinifera* was accompanied by a description of its morphology and uses, which provide vital pieces of information about how Palisot de Beauvois actually saw this species. He describes *R. vinifera* as a “tree of average size” that is “not distinguished by the length its trunk” but by the size of the infructescence (page 77). He suggests that the locals prefer to tap *R. vinifera* (in contrast to *Elaeis guineensis*, "palmiers à vins") because of the "great facility they have to collect it without danger" (page 78). These descriptions suggest that the *R. vinifera* he saw had a very short stem. He goes on to describe the multitude of uses of this species such as the leaves for weaving and thatching, and especially the wine that is tapped from the trunks and even the fruits.

A few years later, in their revision of African palms, Mann and Wendland (1864) suggested that *R. vinifera* was closely related to the South American *R. taedigera* Mart. (von Martius 1823) “in the shape of fruit and scales” (page 423). However, as noted above, the illustration of the fruits (Fig. 1B) are certainly from a different species than the inflorescences. In their description of *R. vinifera* (page 437), Mann and Wendland (1864) cited a specimen collected by *Mann s.n.* from the “Banks of the Old Calabar” as being *R. vinifera*. We were not able to locate this particular specimen (neither in K nor GOET).

Beccari (1910), in his monograph of *Raphia* (in Italian), described the inflorescences and flowers of *R. vinifera* based on the type specimen he saw in the Candolle Herbarium (now merged in the G general collection) citing the “coplanar” nature of the partial inflorescences (page 88). However, he used the fruiting illustration of Palisot de Beauvois (1804) as a reference to describe the fruits (page 90; translated from Italian: “I have not seen the fruits of the authentic specimens of *R. vinifera* of Palisot de Beauvais [Beauvois]; but those figured in the “Flore d'Oware et de Benin", t. 46, f. 1”). He also indicated he had no precise idea about the general aspect of the species (size or trunk). In addition, he recognized that the specimen cited by Mann and Wendland (1864) as *R. vinifera* (*Mann s.n.*, see above) was wrongly identified, and subsequently created a new name to accommodate it (*R. mannii* Becc.). Finally, Beccari (1910) described the species *R. wendlandii* Becc. from a collection of *Mann* s.n. collected in "Fernando Poo", currently the Island of Bioko (Equatorial Guinea). The type of this species name is located at K, in the economic botany section under the number 38686.

**Figure 2.**
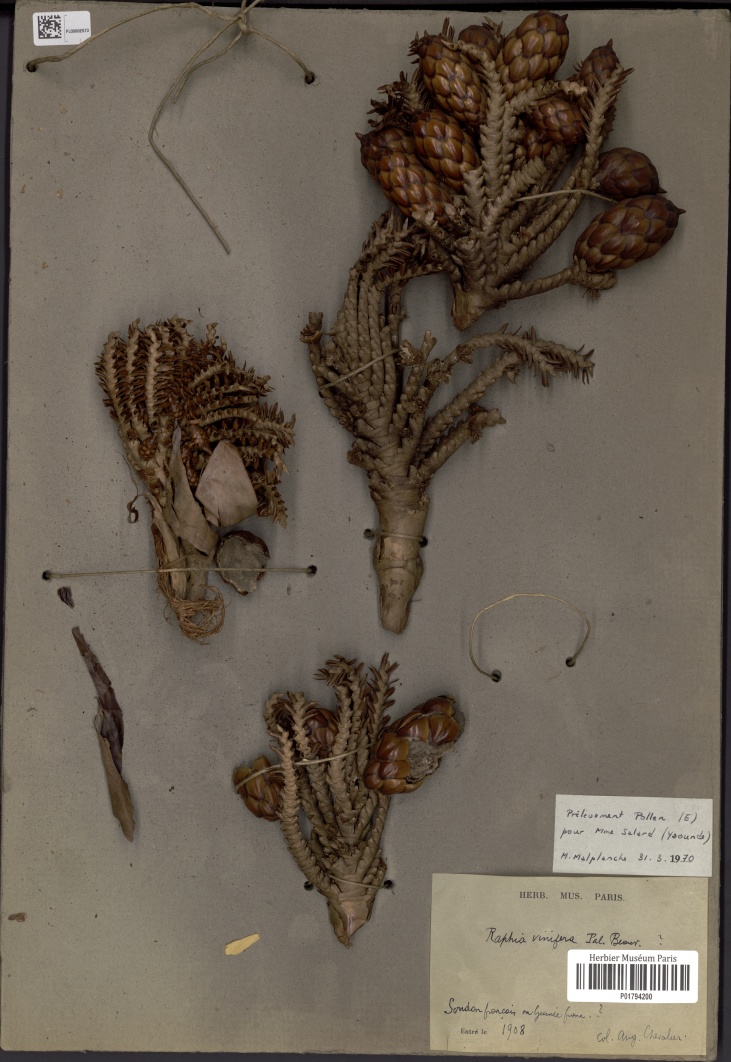
Specimen of *Raphia vinifera* collected by A. Chevalier from Soudan Français or Guinée Française in 1908. [P01794200; https://science.mnhn.fr/institution/mnhn/collection/p/item/p01794200?listIndex=113&listCount=223]. Scanned by the Muséum Nationale d'Histoire Naturelle, Paris.

Chevalier (1932), based on the flowering inflorescence description of Beccari (1910), cited a *Raphia* species occurring in several countries across West Africa (Benin, Burkina Faso, Ivory Coast, Mali), which he tentatively associated to *R. vinifera*. Chevalier (1932) noted that this species was common in valleys of small rivers, was characterized by an absent or very short stem, and was tapped for wine. We saw one of his collections in Paris (P01794200, Fig. 2) from Mali (or Guinée), which contains four partial inflorescences closely matching the type of *R. vinifera* in G (see bleow).

In the first edition of the Flora of Tropical West Africa, Hutchinson and Dalziel (1936) described *R. vinifera* as a "medium sized tree" with a stem longer than 3 m tall (page 387), which contrasts with Palisot de Beauvois’s description (see above). They reproduced the drawing of the flowering inflorescence of Palisot de Beauvois (their Figure 317, page 389) and suggested that a portion of the inflorescence is present in Kew, though we did not locate it. In fact, no collection (collector/number) is cited for this species by Hutchinson and Dalziel (1936).

Confusion really settled in when, in his review of west African *Raphia*’s, Russell (1965) published a long description of *R. vinifera* indicating it has a stem up to 5 m tall, apparently agreeing with Hutchinson and Dalziel (1936) but in contrast with Palisot de Beauvois (1804). He also described the inflorescences: “branchlets are clearly seen to be in 4 ranks extending right round the partial inflorescence” (page 180, accompanied by an illustration of the inflorescence, Figure 7A). The description and illustration clearly correspond to the raphiate type of Otedoh (1982) and do not match the type specimen or the description provided by Palisot de Beauvois (1804). The illustration and descriptions provided by Russell (1965), and in subsequent taxonomic works by the same author (Russell 1968, Tuley and Russell 1966), actually correspond to the name *R. mannii* (see above), which he placed in synonymy with *R. vinifera* in addition to *R. wendlandii* (*Russell 1968*). This publication led most authors, mainly working in Nigeria, into error about the morphology of *R. vinifera*.

**Figure 3.**
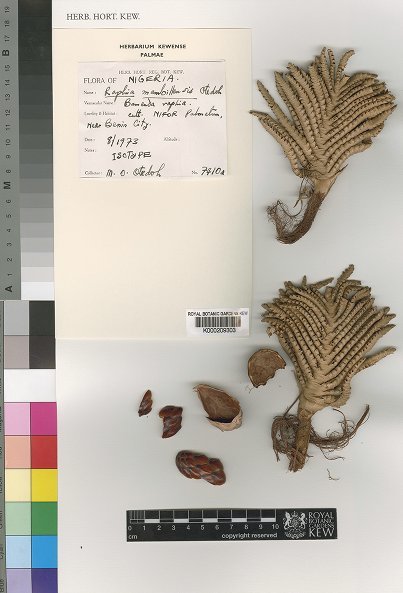
Isotype of *Raphia mambillensis* Otedoh (*Otedoh 7401*; K000209303)

In the latest revision of the genus, Otedoh (1982) placed the name *R. vinifera* in the “raphiate” section, probably following the fruiting description (and illustration) of *R. vinifera (Palisot de Beauvois 1804*). Interestingly, he associated very few uses to the species (“would yield good thatch and bamboos” page 162), suggesting it was sacred and generally protected. In the same publication, Otedoh (1982) described the new species *R. mambillensis* Otedoh (Otedoh 7401; Fig. 3) in the flabellate section (planar shaped partial inflorescences). This species was described as a small *Raphia* palm with a subterranean stem and leaves 5–8 m long arising from the ground. It is a common species mainly growing between 1200 and 2000 m in the Cameroonian Volcanic Line and reported from Nigeria, Cameroon, Central African Republic and South Sudan (Chevalier 1932, Letouzey 1978). Interestingly, Otedoh (1982) also noted that *R. mambillensis* grows alongside streams or in swamps at lower altitudes (page 164). *Raphia mambillensis* is a widely-used palm for wine, thatching and as a source for grubs. Prior to Otedoh (1982), *R. mambillensis* used to be confused with *R. farinifera* (e.g. *Russell 1965*). In addition, Otedoh (1982) also described a new variety: R. vinifera var. nigerica Otedoh, distinct by the symmetrically opposite leafets.

Finally, in an overview of African palms, Tuley (1995), following Tuley and Russell (1966) and Otedoh (1982), placed *R. vinifera* in the raphiate section. He went further and suggested that Palisot de Beauvois (1804) (page 82) got confused with the different palms he saw and that the description of the uses of *R. vinifera* were in fact those of *R. hookeri*, which he refers to as the "true wine palm".

### Type specimen of *Raphia vinifera*

**Figure 4.**
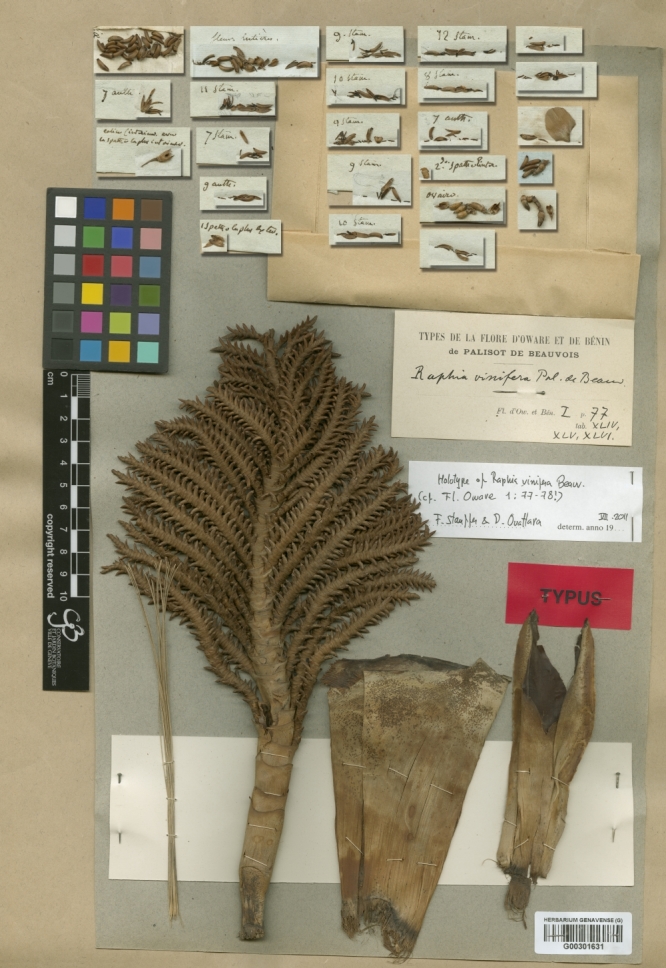
Scan of the lectotype (here designated) of *Raphia vinifera*, stored at the G herbarium [G00301631]. Image scanned by Conservatoire et Jardin Botanique (Geneva).

As indicated above, no holotype was explicitly mentioned in the protologue (Palisot de Beauvois 1804). In his revision of the genus, Beccari (1910) mentions studying a "type exemplar" (page 88) of *R. vinifera* from Palisot de Beauvois deposited at the “De Candolle Herbarium” in Geneva. He does not, however, provide more details about the specimen. In an unpublished PhD thesis, Otedoh (1976) identified Palisot de Beauvois *s.n.* from the districts of Warri and Benin as the type (page 245). Finally, Stauffer et al. (2017), in an overview of taxonomic knowledge on African palms, provided a scan of a Palisot de Beauvois *s.n.* collection from G and suggested it was the holotype of *R. vinifera* (barcode: G00301631, Fig. 4). We have now identified seven specimens belonging to the collection Palisot de Beauvois *s.n.*, with isotypes deposited in five herbaria (see below). This specimen is composed of a partial inflorescence with young flowers and large bracts, belonging to the flabellate type, and is clearly what is illustrated in Fig. 1A of Palisot de Beauvois (1804). To date, we did not find any *Raphia* specimens collected by Palisot de Beauvois with fruits.

A precise understanding of *R. vinifera* remains unclear. From the protologue and the type specimen (Fig. 4), it is clear that the name *R. vinifera* refers to a species with an acaulescent or very short stem, flabellate inflorescences and having multiple uses. It is not a species with a distinct stem, raphiate inflorescences and few uses as suggested by Russell (1965), Otedoh (1982) or Tuley (1995). The flabellate inflorescences as seen on the type specimen of *R. vinifera* (Fig. 4) strongly resemble in shape the morphology of the isotype of *R. mambillensis* (Fig. 3). We thus suggest that the latter is a synonym of the former. In addition, we officially lectotypify the Palisot de Beauvois *s.n.* as the type of the name *R. vinifera*. We place the other specimens as isolectotypes.

The original illustrations of one partial inflorescence and one fruiting inflorescence from two different species have led to confusion (Fig. 1, Palisot de Beauvois 1804). Prior to Russell (1965), most authors agreed that *R. vinifera* had coplanar inflorescences (Beccari 1910, Chevalier 1932) and thus agreed with the description and type of Palisot de Beauvois (1804). It is interesting that Otedoh (1982) published the name *R. mambillensis* having seen the type specimen of *R. vinifera*, despite clear resemblances. Russell (1965), by describing and illustrating *R. vinifera* with a raphiate type inflorescence, created confusion around the proper identity of *R. vinifera*, and the application of the name has since been unclear and inconsistent in local floras or treatments (Akoègninou et al. 2006, Letouzey 1978, Tuley 1995, Stauffer et al. 2014, Otedoh 1982).

However, it remains unclear to what species the illustration of the fruiting partial inflorescence of Palisot de Beauvois (1804) belongs, as well as what Russell (1965) and Otedoh (1982) called *R. vinifera*. The descriptions, illustrations and photographs provided by Russell (1965) and Tuley and Russell (1966) correspond to *R. mannii*, a widespread West African species. Otedoh (1982), nevertheless, considered *R. mannii* as a valid species (including the name *R. wendlandii*, which is a synonym of *R. mannii*). For Otedoh (1982), *R. vinifera* resembles *R. africana* Otedoh, another little-known species. Thus, the morphological concepts of *R. vinifera* of Otedoh (1982) and Russell (1965) are not the same. Knowledge of Nigerian *Raphias* remains complicated and more field work is needed to properly sort out the different species in this region. In addition, we tentatively associate the specimens identified as R. vinifera var. nigerica by Otedoh (1982) with *R. mannii*.

## Taxon treatments

### Raphia vinifera

P.Beauv., 1804

76BF7E96A4865D86A1A202C94015A966

Raphia vinifera *Palisot de Beauvois s.n.*
Raphia mambillensis Otedoh, 1982 - Otedoh (1982): 163 **syn. n.**
Raphia vinifera *M.O. Otedoh 7401*

#### Materials

- **Type status:** Other material. **Occurrence:** recordNumber: Keay, R.W.J. 37938; recordedBy: Keay, R.W.J.; lifeStage: adult; preparations: Native; **Taxon:** taxonID: urn:lsid:ipni.org:names:669541-1; scientificName: Raphia vinifera; kingdom: Plantae; class: Magnoliopsida ; order: Arecales; family: Arecaceae; genus: Raphia; specificEpithet: vinifera; scientificNameAuthorship: P.Beauv.; **Location:** continent: Africa; country: Cameroon; stateProvince: North-West Region; locality: About 1 mile from Bamenda on the Santa road; decimalLatitude: 5.933330; decimalLongitude: 10.166700; geodeticDatum: WGS84; **Identification:** identifiedBy: Mogue Kamga, S.; dateIdentified: 2019; **Event:** eventDate: 1960-5-10; year: 1960; month: 5; day: 10; **Record Level:** language: english; collectionID: urn:lsid:biocol.org:col:34252; basisOfRecord: PreservedSpecimen
- **Type status:** Other material. **Occurrence:** recordNumber: Brunt, M.A. 1140 A; recordedBy: Brunt, M.A.; lifeStage: adult; preparations: Native; **Taxon:** taxonID: urn:lsid:ipni.org:names:669541-1; scientificName: Raphia vinifera; kingdom: Plantae; class: Magnoliopsida ; order: Arecales; family: Arecaceae; genus: Raphia; specificEpithet: vinifera; scientificNameAuthorship: P.Beauv.; **Location:** continent: Africa; country: Cameroon; stateProvince: North-West Region; locality: Baforkum village on road up to Bambui Experimental Station. Mt Baku.; decimalLatitude: 6.050000; decimalLongitude: 10.233300; geodeticDatum: WGS84; **Identification:** identifiedBy: Mogue Kamga, S.; dateIdentified: 2019; **Event:** eventDate: 1963-6-15; year: 1963; month: 6; day: 15; **Record Level:** language: english; collectionID: urn:lsid:biocol.org:col:34252; basisOfRecord: PreservedSpecimen
- **Type status:** Other material. **Occurrence:** recordNumber: Brunt, M.A. 1195; recordedBy: Brunt, M.A.; lifeStage: adult; preparations: Native; **Taxon:** taxonID: urn:lsid:ipni.org:names:669541-1; scientificName: Raphia vinifera; kingdom: Plantae; class: Magnoliopsida ; order: Arecales; family: Arecaceae; genus: Raphia; specificEpithet: vinifera; scientificNameAuthorship: P.Beauv.; **Location:** continent: Africa; country: Cameroon; stateProvince: North-West Region; locality: Baba road; decimalLatitude: 6.000000; decimalLongitude: 10.500000; geodeticDatum: WGS84; **Identification:** identifiedBy: Mogue Kamga, S.; dateIdentified: 2019; **Event:** eventDate: 1963-7-18; year: 1963; month: 7; day: 18; **Record Level:** language: english; collectionID: urn:lsid:biocol.org:col:34252; basisOfRecord: PreservedSpecimen
- **Type status:** Other material. **Occurrence:** recordNumber: Fay, J.M. 2987; recordedBy: Fay, J.M.; lifeStage: adult; preparations: Native; **Taxon:** taxonID: urn:lsid:ipni.org:names:669541-1; scientificName: Raphia vinifera; kingdom: Plantae; class: Magnoliopsida ; order: Arecales; family: Arecaceae; genus: Raphia; specificEpithet: vinifera; scientificNameAuthorship: P.Beauv.; **Location:** continent: Africa; country: Central African Republic; stateProvince: Bamingui-Bangoran; locality: Manovo-Gounda St floris National park, camp Koumbala; decimalLatitude: 8.48583; decimalLongitude: 21.2025; geodeticDatum: WGS84; **Identification:** identifiedBy: Dransfield, J.; dateIdentified: 1987; **Event:** eventDate: 1982-10-8; year: 1982; month: 10; day: 8; **Record Level:** language: english; collectionID: urn:lsid:biocol.org:col:34252; basisOfRecord: PreservedSpecimen
- **Type status:** Other material. **Occurrence:** recordNumber: Otedoh, M.O. n.s.; recordedBy: Otedoh, M.O.; lifeStage: adult; preparations: Native; **Taxon:** taxonID: urn:lsid:ipni.org:names:669541-1; scientificName: Raphia vinifera; kingdom: Plantae; class: Magnoliopsida ; order: Arecales; family: Arecaceae; genus: Raphia; specificEpithet: vinifera; scientificNameAuthorship: P.Beauv.; **Location:** continent: Africa; country: Nigeria; stateProvince: Edo State; locality: NIFOR plantation near Benin city; decimalLatitude: 6.390920; decimalLongitude: 5.581782; geodeticDatum: WGS84; **Identification:** identifiedBy: Mogue Kamga, S.; dateIdentified: 2019; **Event:** eventDate: 1973-8-0; year: 1973; month: 8; **Record Level:** language: english; collectionID: urn:lsid:biocol.org:col:34252; basisOfRecord: PreservedSpecimen
- **Type status:** Other material. **Occurrence:** recordNumber: Brunt, M.A. 1197; recordedBy: Brunt, M.A.; lifeStage: adult; preparations: Native; **Taxon:** taxonID: urn:lsid:ipni.org:names:669541-1; scientificName: Raphia vinifera; kingdom: Plantae; class: Magnoliopsida ; order: Arecales; family: Arecaceae; genus: Raphia; specificEpithet: vinifera; scientificNameAuthorship: P.Beauv.; **Location:** continent: Africa; country: Cameroon; stateProvince: North-West Region; locality: Baba road; decimalLatitude: 6.048130; decimalLongitude: 10.459200; geodeticDatum: WGS84; **Identification:** identifiedBy: Mogue Kamga, S.; dateIdentified: 2019; **Event:** eventDate: 1963-7-18; year: 1963; month: 7; day: 18; **Record Level:** language: english; collectionID: urn:lsid:biocol.org:col:34252; basisOfRecord: PreservedSpecimen
- **Type status:** Other material. **Occurrence:** recordNumber: Brunt, M.A. 842; recordedBy: Brunt, M.A.; lifeStage: adult; preparations: Native; **Taxon:** taxonID: urn:lsid:ipni.org:names:669541-1; scientificName: Raphia vinifera; kingdom: Plantae; class: Magnoliopsida ; order: Arecales; family: Arecaceae; genus: Raphia; specificEpithet: vinifera; scientificNameAuthorship: P.Beauv.; **Location:** continent: Africa; country: Cameroon; stateProvince: North-West Region; locality: Baba road; decimalLatitude: 6.048130; decimalLongitude: 10.459200; geodeticDatum: WGS84; **Identification:** identifiedBy: Mogue Kamga, S.; dateIdentified: 2018; **Event:** eventDate: 1962-0-0; year: 1962; **Record Level:** language: english; collectionID: urn:lsid:biocol.org:col:34252; basisOfRecord: PreservedSpecimen
- **Type status:** Other material. **Occurrence:** recordNumber: Brunt, M.A. 1196; recordedBy: Brunt, M.A.; lifeStage: adult; preparations: Native; **Taxon:** taxonID: urn:lsid:ipni.org:names:669541-1; scientificName: Raphia vinifera; kingdom: Plantae; class: Magnoliopsida ; order: Arecales; family: Arecaceae; genus: Raphia; specificEpithet: vinifera; scientificNameAuthorship: P.Beauv.; **Location:** continent: Africa; country: Cameroon; stateProvince: North-West Region; locality: Baba road; decimalLatitude: 6.048130; decimalLongitude: 10.459200; geodeticDatum: WGS84; **Identification:** identifiedBy: Mogue Kamga, S.; dateIdentified: 2018; **Event:** eventDate: 1963-7-18; year: 1963; month: 7; day: 18; **Record Level:** language: english; collectionID: urn:lsid:biocol.org:col:34252; basisOfRecord: PreservedSpecimen
- **Type status:** Other material. **Occurrence:** recordNumber: Brunt, M.A. 1139; recordedBy: Brunt, M.A.; lifeStage: adult; preparations: Native; **Taxon:** taxonID: urn:lsid:ipni.org:names:669541-1; scientificName: Raphia vinifera; kingdom: Plantae; class: Magnoliopsida ; order: Arecales; family: Arecaceae; genus: Raphia; specificEpithet: vinifera; scientificNameAuthorship: P.Beauv.; **Location:** continent: Africa; country: Cameroon; stateProvince: North-West Region; locality: Baforkum village, by bridge on road up to Bambui Experimental Station. Mt Baku; decimalLatitude: 6.048975; decimalLongitude: 10.234535; geodeticDatum: WGS84; **Identification:** identifiedBy: Mogue Kamga, S.; dateIdentified: 2018; **Event:** eventDate: 1963-6-15; year: 1963; month: 6; day: 15; **Record Level:** language: english; collectionID: urn:lsid:biocol.org:col:34252; basisOfRecord: PreservedSpecimen
- **Type status:** Other material. **Occurrence:** recordNumber: Brunt, M.A. 1194; recordedBy: Brunt, M.A.; lifeStage: adult; preparations: Native; **Taxon:** taxonID: urn:lsid:ipni.org:names:669541-1; scientificName: Raphia vinifera; kingdom: Plantae; class: Magnoliopsida ; order: Arecales; family: Arecaceae; genus: Raphia; specificEpithet: vinifera; scientificNameAuthorship: P.Beauv.; **Location:** continent: Africa; country: Cameroon; stateProvince: North-West Region; locality: Ndop plain, Ndop-Baba road; decimalLatitude: 6.048130; decimalLongitude: 10.459200; geodeticDatum: WGS84; **Identification:** identifiedBy: Mogue Kamga, S.; dateIdentified: 2018; **Event:** eventDate: 1963-7-18; year: 1963; month: 7; day: 18; **Record Level:** language: english; collectionID: urn:lsid:biocol.org:col:34252; basisOfRecord: PreservedSpecimen
- **Type status:** Other material. **Occurrence:** recordNumber: Palisot de Beauvois, A.M.F.J. n.s.; recordedBy: Palisot de Beauvois, A.M.F.J.; lifeStage: adult; preparations: Native; **Taxon:** taxonID: urn:lsid:ipni.org:names:669541-1; scientificName: Raphia vinifera; kingdom: Plantae; class: Magnoliopsida ; order: Arecales; family: Arecaceae; genus: Raphia; specificEpithet: vinifera; scientificNameAuthorship: P.Beauv.; **Location:** continent: Africa; country: Nigeria; stateProvince: Edo State; decimalLatitude: 5.51666666666667; decimalLongitude: 5.75; geodeticDatum: WGS84; **Identification:** identifiedBy: Stauffer, F.W.; dateIdentified: 2011; **Event:** eventDate: 1813-6-0; year: 1813; month: 6; **Record Level:** language: english; collectionID: urn:lsid:biocol.org:col:34252; basisOfRecord: PreservedSpecimen
- **Type status:** Other material. **Occurrence:** recordNumber: Jacques-Félix, H. 3089; recordedBy: Jacques-Félix, H.; lifeStage: adult; preparations: Native; **Taxon:** taxonID: urn:lsid:ipni.org:names:669541-1; scientificName: Raphia vinifera; kingdom: Plantae; class: Magnoliopsida ; order: Arecales; family: Arecaceae; genus: Raphia; specificEpithet: vinifera; scientificNameAuthorship: P.Beauv.; **Location:** continent: Africa; country: Cameroon; stateProvince: West Region; locality: Bayangam; decimalLatitude: 5.289809; decimalLongitude: 10.420059; geodeticDatum: WGS84; **Identification:** identifiedBy: Mogue Kamga, S.; dateIdentified: 2018; **Event:** eventDate: 1938-1-0; year: 1938; month: 1; **Record Level:** language: english; collectionID: urn:lsid:biocol.org:col:34252; basisOfRecord: PreservedSpecimen
- **Type status:** Other material. **Occurrence:** recordNumber: Fotius, G. 3088; recordedBy: Fotius, G.; lifeStage: adult; preparations: Native; **Taxon:** taxonID: urn:lsid:ipni.org:names:669541-1; scientificName: Raphia vinifera; kingdom: Plantae; class: Magnoliopsida ; order: Arecales; family: Arecaceae; genus: Raphia; specificEpithet: vinifera; scientificNameAuthorship: P.Beauv.; **Location:** continent: Africa; country: Cameroon; stateProvince: Adamawa Region; locality: Lompta, 38 km SSW Tignčre; decimalLatitude: 7.368999; decimalLongitude: 12.650306; geodeticDatum: WGS84; **Identification:** identifiedBy: Mogue Kamga, S.; dateIdentified: 2019; **Event:** eventDate: 1979-3-14; year: 1979; month: 3; day: 14; **Record Level:** language: english; collectionID: urn:lsid:biocol.org:col:34252; basisOfRecord: PreservedSpecimen
- **Type status:** Other material. **Occurrence:** recordNumber: Chevalier, A.J.B. 7701; recordedBy: Chevalier, A.J.B.; lifeStage: adult; preparations: Native; **Taxon:** taxonID: urn:lsid:ipni.org:names:669541-1; scientificName: Raphia vinifera; kingdom: Plantae; class: Magnoliopsida ; order: Arecales; family: Arecaceae; genus: Raphia; specificEpithet: vinifera; scientificNameAuthorship: P.Beauv.; **Location:** continent: Africa; country: Central African Republic; stateProvince: Nana-Mambéré; locality: Dar Rounga, Kounde; decimalLatitude: 6.116670; decimalLongitude: 14.633300; geodeticDatum: WGS84; **Identification:** identifiedBy: Mogue Kamga, S.; dateIdentified: 2019; **Event:** eventDate: 1903-3-0; year: 1903; month: 3; **Record Level:** language: english; collectionID: urn:lsid:biocol.org:col:34252; basisOfRecord: PreservedSpecimen
- **Type status:** Other material. **Occurrence:** recordNumber: Chevalier, A.J.B. 8397; recordedBy: Chevalier, A.J.B.; lifeStage: adult; preparations: Native; **Taxon:** taxonID: urn:lsid:ipni.org:names:669541-1; scientificName: Raphia vinifera; kingdom: Plantae; class: Magnoliopsida ; order: Arecales; family: Arecaceae; genus: Raphia; specificEpithet: vinifera; scientificNameAuthorship: P.Beauv.; **Location:** continent: Africa; country: Central African Republic; stateProvince: Bamingui-Bangoran; locality: Chari oriental: Kouti et pays Ndouka, Télé; decimalLatitude: 8.863486; decimalLongitude: 20.927135; geodeticDatum: WGS84; **Identification:** identifiedBy: Mogue Kamga, S.; dateIdentified: 2019; **Event:** eventDate: 1903-5-12; year: 1903; month: 5; day: 12; **Record Level:** language: english; collectionID: urn:lsid:biocol.org:col:34252; basisOfRecord: PreservedSpecimen
- **Type status:** Other material. **Occurrence:** recordNumber: Jacques-Félix, H. 8806; recordedBy: Jacques-Félix, H.; lifeStage: adult; preparations: Native; **Taxon:** taxonID: urn:lsid:ipni.org:names:669541-1; scientificName: Raphia vinifera; kingdom: Plantae; class: Magnoliopsida ; order: Arecales; family: Arecaceae; genus: Raphia; specificEpithet: vinifera; scientificNameAuthorship: P.Beauv.; **Location:** continent: Africa; country: Cameroon; stateProvince: Adamawa Region; locality: E. Adamawa, Hossere Sille, N. Of Meiganga; decimalLatitude: 6.516670; decimalLongitude: 14.300000; geodeticDatum: WGS84; **Identification:** identifiedBy: Mogue Kamga, S.; dateIdentified: 2019; **Event:** eventDate: 1967-10-22; year: 1967; month: 10; day: 22; **Record Level:** language: english; collectionID: urn:lsid:biocol.org:col:34252; basisOfRecord: PreservedSpecimen
- **Type status:** Other material. **Occurrence:** recordNumber: Chevalier, A.J.B. n.s.; recordedBy: Chevalier, A.J.B.; lifeStage: adult; preparations: Native; **Taxon:** taxonID: urn:lsid:ipni.org:names:669541-1; scientificName: Raphia vinifera; kingdom: Plantae; class: Magnoliopsida ; order: Arecales; family: Arecaceae; genus: Raphia; specificEpithet: vinifera; scientificNameAuthorship: P.Beauv.; **Location:** continent: Africa; country: Mali; geodeticDatum: WGS84; **Identification:** identifiedBy: Mogue Kamga, S.; dateIdentified: 2019; **Event:** eventDate: 1908-0-0; year: 1908; **Record Level:** language: english; collectionID: urn:lsid:biocol.org:col:34252; basisOfRecord: PreservedSpecimen
- **Type status:** Other material. **Occurrence:** recordNumber: Vaillant, A. 1095; recordedBy: Vaillant, A.; lifeStage: adult; preparations: Native; **Taxon:** taxonID: urn:lsid:ipni.org:names:669541-1; scientificName: Raphia vinifera; kingdom: Plantae; class: Magnoliopsida ; order: Arecales; family: Arecaceae; genus: Raphia; specificEpithet: vinifera; scientificNameAuthorship: P.Beauv.; **Location:** continent: Africa; country: Cameroon; stateProvince: West Region; locality: Dschang; decimalLatitude: 5.457084; decimalLongitude: 10.062661; geodeticDatum: WGS84; **Identification:** identifiedBy: Mogue Kamga, S.; dateIdentified: 2019; **Event:** eventDate: 1946-3-0; year: 1946; month: 3; **Record Level:** language: english; collectionID: urn:lsid:biocol.org:col:34252; basisOfRecord: PreservedSpecimen
- **Type status:** Other material. **Occurrence:** recordNumber: Couvreur, T.L.P. 638; recordedBy: Couvreur, T.L.P.; lifeStage: adult; preparations: Native; **Taxon:** taxonID: urn:lsid:ipni.org:names:669541-1; scientificName: Raphia vinifera; kingdom: Plantae; class: Magnoliopsida ; order: Arecales; family: Arecaceae; genus: Raphia; specificEpithet: vinifera; scientificNameAuthorship: P.Beauv.; **Location:** continent: Africa; country: Cameroon; stateProvince: North-West Region; locality: Belon, Zwinkles Guest House, in front of bathrooms.; decimalLatitude: 6.191145; decimalLongitude: 10.382481; geodeticDatum: WGS84; **Identification:** identifiedBy: Couvreur, T.L.P.; dateIdentified: 2014; **Event:** eventDate: 2014-3-5; year: 2014; month: 3; day: 5; **Record Level:** language: english; collectionID: urn:lsid:biocol.org:col:34252; basisOfRecord: PreservedSpecimen
- **Type status:** Other material. **Occurrence:** recordNumber: Couvreur, T.L.P. 640; recordedBy: Couvreur, T.L.P.; lifeStage: adult; preparations: Native; **Taxon:** taxonID: urn:lsid:ipni.org:names:669541-1; scientificName: Raphia vinifera; kingdom: Plantae; class: Magnoliopsida ; order: Arecales; family: Arecaceae; genus: Raphia; specificEpithet: vinifera; scientificNameAuthorship: P.Beauv.; **Location:** continent: Africa; country: Cameroon; stateProvince: North-West Region; locality: Belon, on small path around Zwinkles Guest House; decimalLatitude: 6.191145; decimalLongitude: 10.382481; geodeticDatum: WGS84; **Identification:** identifiedBy: Couvreur, T.L.P.; dateIdentified: 2014; **Event:** eventDate: 2014-3-5; year: 2014; month: 3; day: 5; **Record Level:** language: english; collectionID: urn:lsid:biocol.org:col:34252; basisOfRecord: PreservedSpecimen
- **Type status:** Other material. **Occurrence:** recordNumber: Hoyle, A.C. 491; recordedBy: Hoyle, A.C.; lifeStage: adult; preparations: Native; **Taxon:** taxonID: urn:lsid:ipni.org:names:669541-1; scientificName: Raphia vinifera; kingdom: Plantae; class: Magnoliopsida ; order: Arecales; family: Arecaceae; genus: Raphia; specificEpithet: vinifera; scientificNameAuthorship: P.Beauv.; **Location:** continent: Africa; country: Sudan; stateProvince: Lol State; locality: Boro River, W and N.W. of Sa'id Bundas; decimalLatitude: 8.505848; decimalLongitude: 24.669303; geodeticDatum: WGS84; **Identification:** identifiedBy: Mogue Kamga, S.; dateIdentified: 2019; **Event:** eventDate: 1939-1-22; year: 1939; month: 1; day: 22

#### Description

**Figure 5.**
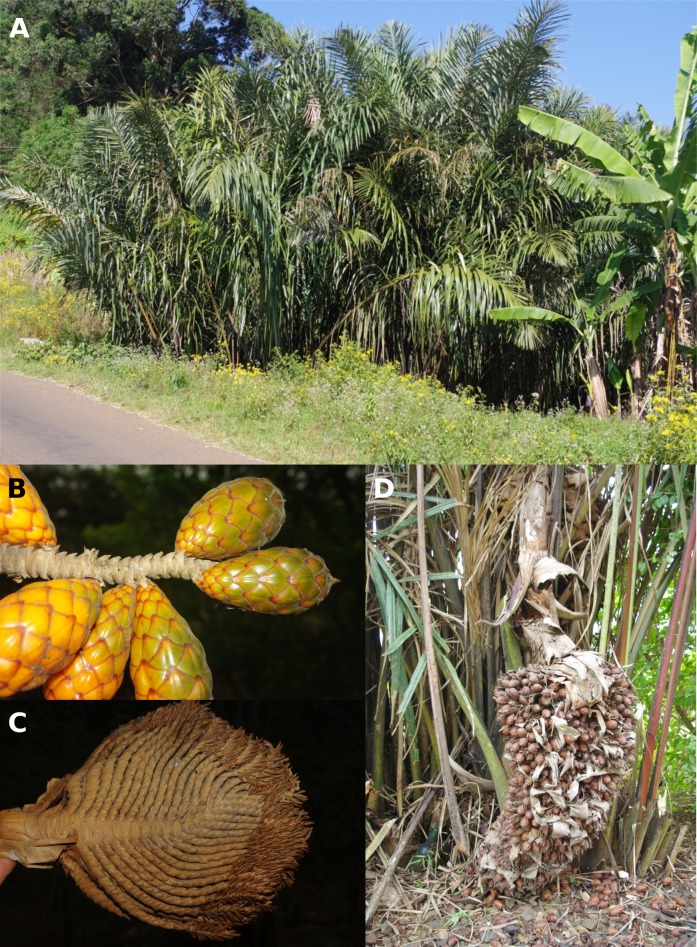
*Raphia vinifera* in Cameroon. **A** Habit along road near Oku (North West region) **B** Fruits **C** Inflorescence with old male flowers at apex. Note flabellate nature **D** Inflorescences, note acaulescent habit of palm and the large covering bracts. (Photos Thomas L.P. Couvreur, B-D: Couvreur 638 (WAG,YA)).

Acaulescent palm 7–10 m high overall (including leaves), clustering; dead leaf sheaths persistent, remains of leaf bases near the ground formed through disintegration of leaf sheath, flat, scaly, dark brown. ***Leaves*** 10–12, 7–10 m long in total, arising directly from the ground, vertically and then arched downwards towards apex; ***sheath*** 80–90 cm long, ***petiole*** 3–5 m long; ***rachis*** 4–6 m long; ***pinnae*** 100–126 per side, 0.2–1.3 m long, 5–55 mm wide, irregularly arranged in 4 planes, arching downwards towards the apex, midrib and pinnae margins armed with spines 1–2 mm long but very few to absent on older leaves. Leaves subtending inflorescence reduced. ***Inflorescences*** 3–4(–5), pendulous or semi pendulous (nodding), 0.60–1.95 m long in total; young inflorescences light green to purple green, older ones light brown to grey-brown; ***rachis*** 0.45–1.20 m long, bearing numerous pronounced bracts rarely empty, almost completely covering the inflorescence, pinkish-brown abaxially (young) to light brown (older); ***rachillae*** in two orders: ***first order branches*** 50–60, 6–18 cm long, flabellate shaped, dorsi ventrally compressed, alternating in 2 rows on each side of the rachis, smooth; ***second order branches*** 60–64, 4–12 cm long, dorsi ventrally compressed, alternating in 2 rows on each side of first order rachillae, bud flattened, smooth; ***Flowers*** solitary, exerted, inserted in one row, rarely two on each side of second order rachillae, staminate flowers distal, pistillate flowers basal. ***Staminate flower: calyx*** fused, tubular, bearing three shallow lobes; ***corolla*** comprising 3 petals, 7–10 mm long, 2.5–3 mm wide, basally connate for 1/3 of their length, oblong, apex acuminate, margins entire, smooth, stiff; ***stamens*** 6–8. ***Pistillate flowers***: ***calyx*** fused, tubular, bearing three very shallow lobes; ***corolla*** comprising 3 petals, 2–4 mm long, separate, basely attached, 3 pointed tips, acuminate, margins irregular, smooth; ***staminodial ring*** with 6–7 staminodes, 0.5–2 mm long, fused between them, adnate to petals for ca. 1 mm; anthers sagittate, 0.2–1.5 mm long; ***gynoecium*** 3–5.5 mm long, 1.5–2 mm wide, ovary ca. 2.8–5.3 mm, 1.3–1.9 mm wide, ovoid to ovate long, completely covered with scales, developing at 3/4 height of the gynoecium, larger scales at mid portion to base; style absent or very short; stigma ca. 1 mm long, papillae not observed (flowers young). ***Fruits*** ellipsoid, oblong to obovoid, 6–9 cm long, 2.5 (young) –5 (mature) cm large, pointed beak 3.7–4.5 mm long, ca. 3 mm wide; (usually wider towards the beak), scales 8–10 rows (usually 9); seed 1 oblong, 2.5–7 cm long, 2–3 cm wide, with ruminated endosperm (Fig. 5).

#### Distribution

**Figure 6.**
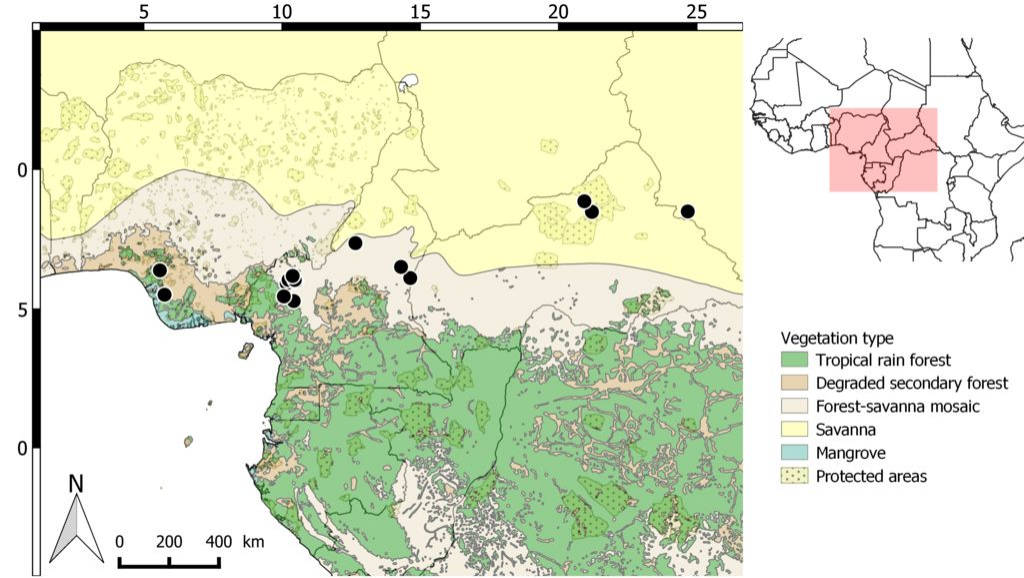
Distribution of *Raphia vinifera* based on documented herbarium specimens.

*Raphia vinifera* is mainly distributed in the Northwest, West and Adamaoua regions of Cameroon, where it is very abundant and even cultivated. Fewer botanical collections are known from the Delta state of Nigeria, Central African Republic and South Sudan (Fig. 6). One specimen from August Chevalier (*Chevalier s.n.*, 1908 [P01794200], see Fig. 2) is marked as “Soudan Français [now Mali] ou Guinée Française?”. Chevalier (1932) documents the presence of *R. vinifera* in “Soudan Français”, so it is possible that this specimen is from Mali. We were not able to geolocate this specimen, although it seems possible it was collected close to Bamako (Kita). The literature also documents the presence of this species in several other West African regions or countries for which we did not locate specimens: the Mambillen Mountains in the Gongola state in south-eastern Nigeria (Otedoh 1982, Tuley 1995), Mali (Bamako, Kita), Burkina Faso (Sikasso, Bobo-Dioulasso), Ivory Coast, Benin [Bas-Dahomey] (Chevalier 1932, page 209). The Flore Analytique du Bénin (Akoègninou et al. 2006) mentions three species of *Raphia* including *R. vinifera*. However, this is clearly the “vinifera” as described by Otedoh (1982) and Tuley (1995), with a “trunk 6-10 m” (translated from French, page 60) and not the one we describe here. This description does not correspond to *R. vinifera* as described here and no acaulescent *Raphia* is mentioned. In addition, recent field trips by colleagues did not document *R. vinifera* in Ivory Coast (F. Stauffer, pers. com.), Burkina Faso (F. Stauffer, pers. com.) and Benin (V. Salako, pers. com.). Thus, although the distribution of *R. vinifera* in Central Africa is quite clear, its presence needs to be properly documented with recent botanical collections from West Africa.

#### Ecology

The yet uncertain knowledge of *Raphia vinifera*’s distribution leads to an incomplete understanding of its ecology. The species occurs mainly in the transition zone between lower Guinea and the Guineo-Sudanian bioregion in the western highlands of Cameroon and the Guineo-Sudanian bioregion (Fig. 6). The species mainly occurs in open habitats, growing along streams and generally forming monodominant stands. It is cultivated in the West and North West regions of Cameroon where it occurs between 1500 m and 1800 m, reaching 2000 m. In West Africa, *R. vinifer*a is documented from sea level to 1400 m. The presence of the type specimen in the lowland Delta State in Nigeria suggests that *R. vinifera* is not a strict mountain species. However, more field studies in West Africa are needed to precisely document the ecological characteristics of this species.

#### Conservation

The IUCN Red List documents both species, *R. vinifera* and *R. mambillensis*, as Least Concern (LC, Cosiaux et al. 2018). With our new circumscription of *R. vinifera* (which inlcudes the name *R. mambillensis*), this status will probably remain unchanged, although a new full assessement should be conducted. More data are needed on its distribution and ecology from West Africa, including recent collections from other countries outside of Cameroon.

#### Notes

*Raphia vinifera* belongs to the flabellate section as defined by Otedoh (1982) and not to the raphiate one as suggested by Otedoh (1982) and Tuley (1995). In addition, it does not correspond to the descriptions or illustrations provided by Russell (1965), Tuley and Russell (1966) or Russell (1968). In these later publications, *R. vinifera* is confused with *R. mannii*.

*Raphia vinifera* is an acaulescent palm, with planar, characteristic fan-shaped partial inflorescences (Figs 4, 5), with very prominent bracts completely or partially covering the partial inflorescence. The only other species with this type of inflorescence is the widespread *R. farinifera* (Otedoh 1982). The only other species with an acaulescent stem is *R. regalis* Becc., a *tierra firme* species with erect inflorescences (Tuley 1995, Stauffer et al. 2014).

Numerous names have been suggested as synonyms of *R. vinifera* (e.g. *R. mannii*, *R. wendlandii*, *R. diasticha* Burret), but these are not related to the species we describe here.

Fred Stauffer indicates that the specimen conserved at the M herbarium (M0208480, information on specimen) was made by extracting a few rachillea from the holotype in G.

## Supplementary Material

XML Treatment for Raphia vinifera
